# E-Cigarette Use and Lung Cancer Screening Uptake

**DOI:** 10.1001/jamanetworkopen.2024.19648

**Published:** 2024-07-02

**Authors:** Qian Wang, Changchuan Jiang, Melinda L. Hsu, Juan Wisnivesky, Afshin Dowlati, Paolo Boffetta, Chung Yin Kong

**Affiliations:** 1 University Hospitals Seidman Cancer Center, Cleveland, Ohio; 2 Division of Solid Tumor Oncology, Case Western Reserve University, Cleveland, Ohio; 3 Division of Hematology and Oncology, Department of Internal Medicine, University of Texas Southwestern Medical Center, Dallas; 4 Division of General Internal Medicine, Icahn School of Medicine at Mount Sinai, New York, New York; 5 Division of Pulmonary, Critical Care and Sleep Medicine, Icahn School of Medicine at Mount Sinai, New York, New York; 6 Department of Family, Population and Preventive Medicine, Stony Brook University, Stony Brook, New York; 7 Department of Medical and Surgical Sciences, University of Bologna, Bologna, Italy

## Abstract

This cross-sectional study examines e-cigarette use and lung cancer screening uptake among individuals in the US.

## Introduction

Electronic cigarettes (e-cigarettes) have gained popularity since 2007 and have become the second most commonly used tobacco product among US adults in 2021.^1^ E-cigarettes have been increasingly used as tobacco cessation aids.^2,3^ However, there are concerns about the potential long-term cancer risks associated with e-cigarette use.^4^ Since the recommendations from the US Preventive Services Task Force (USPSTF) endorsing lung cancer screening (LCS) in 2013 (revised in 2021), uptake has increased but remains low.^5,6^ The association between e-cigarette use and LCS uptake remains unknown.

## Methods

This cross-sectional study followed the Strengthening the Reporting of Observational Studies in Epidemiology (STROBE) reporting guideline and was exempt for human participants review from the institutional review board at University Hospitals. Informed consent was not required because data were deidentified. Individuals who ever smoked cigarettes and were eligible for LCS according to the USPSTF 2021 recommendations (ie, age 50 to 80 years, 20 pack-years or more smoking history, currently smoking or quit 15 years ago or less) were selected from the 2022 Behavioral Risk Factor Surveillance System (BRFSS) (eMethods and eTable in Supplement 1).^5^ The χ^2^ (for categorical variables) and Kruskal-Wallis (for continuous variables) tests were used to compare baseline demographic characteristics between individuals who underwent LCS vs did not undergo LCS. Logistic regression was used to calculate the association between LCS uptake and e-cigarette use, after adjusting for potential confounders. We also stratified LCS uptake by time since last LCS—ever having an LCS vs having an up-to-date LCS (uptake within the past 1 year). Analyses were weighted per the BRFSS guidelines using SAS version 9.4 (SAS Institute) and R version 2024.04.1 (R Project for Statistical Computing). The significance level was set at 2-sided α < 0.05.

## Results

Of 22 713 eligible individuals, the median (IQR) age was 62 (56-67) years and the sample (weighted percentage) included 12 198 males (56.4%), 19 136 non-Hispanic White individuals (77.7%), 1239 non-Hispanic Black individuals (8.6%), 6122 individuals (26.7%) who underwent LCS, and 3472 individuals (14.6%) who were up-to-date with LCS testing (Table 1). Individuals who underwent LCS were older, more likely to have lower income, comorbidities, had a routine check-up last year, reported poor general health, resided in the Northeast region of the US, and were less likely to be uninsured. They also had a higher pack-year of smoking, had recently quit smoking (individuals who previously used cigarettes), had attempted to quit in the past year (individuals who currently use cigarettes), and were more likely to report never using e-cigarettes compared with those who did not undergo LCS (Table 1). Overall, individuals currently using e-cigarettes had 21% lower odds of having undergone LCS (odds ratio [OR], 0.79; 95% CI, 0.62-1.00) compared with individuals who never used e-cigarettes after adjusting for confounders, with similar trends found in individuals who previously used combustible cigarettes (OR, 0.73; 95% CI, 0.52-1.04) (Table 2). After stratifying by time since last LCS, individuals currently using e-cigarettes had 33% lower odds of being up-to-date with LCS (OR, 0.67; 95% CI, 0.51-0.88) than individuals who never use e-cigarettes (Table 2). Similar findings were seen among individuals who previously used combustible cigarettes and currently use e-cigarettes; they had 46% lower odds of being up-to-date with LCS (OR, 0.54; 95% CI, 0.37-0.80) (Table 2).

**Table 1.** Demographics and Lung Cancer Risk Factors Among Participants Who Were Eligible for LCS, and by LCS Uptake (2022 BRFSS)

Characteristics	Participants, No. (weighted %)			*P* value
	Eligible for LCS (n = 22 713)	Not screened for LC (n = 16 591)	Screened for LC (n = 6122)^a^	
Weighted No. (%)	10 418 903 (100)	7 639 021 (73.3)	2 779 882 (26.7)	NA
Age, median, IQR, y^b^	62 (56-67)	61 (55-66)	64 (59-70)	<.001
Sex				
Male	12 198 (56.4)	8730 (55.8)	3468 (58.0)	.15
Female	10515 (43.6)	7861 (44.2)	2654 (42.0)	
Race and ethnicity				
Hispanic	869 (6.8)	658 (6.9)	211 (6.6)	.42
Non-Hispanic Black	1239 (8.6)	859 (8.2)	380 (9.7)	
Non-Hispanic White	19136 (77.7)	13976 (77.9)	5160 (77.1)	
Other^c^	1469 (6.9)	1098 (7.1)	371 (6.6)	
Annual household income, $				
<50 000	12 043 (50.1)	8651 (48.6)	3392 (54.3)	<.001
≥50 000	7689 (35.8)	5698 (36.9)	1991 (32.7)	
Unknown	2981 (14.1)	2242 (14.5)	739 (13.0)	
Education				
≤High school	10650 (53.9)	7895 (54.7)	2755 (51.5)	.09
Some college	7536 (33.3)	5471 (32.5)	2065 (35.3)	
≥College	4527 (12.9)	3225 (12.8)	1302 (13.1)	
Insurance type^d^				
Any private (<65 y)	5457 (28.0)	4423 (30.9)	1034 (20.1)	<.001
Public (<65 y)	5608 (27.9)	4229 (28.8)	1379 (25.5)	
Medicare (≥65 y)	7442 (28.2)	4858 (23.9)	2584 (40.1)	
Any private (≥65 y)	1122 (4.5)	761 (4.2)	361 (5.5)	
Other public (≥65 y)	1314 (5.3)	816 (4.8)	498 (6.7)	
Uninsured	1139 (6.0)	1027 (7.4)	112 (2.1)	
Cigarette smoking status (combustible)				
Current	13488 (59.4)	9885 (59.3)	3603 (59.6)	.83
Former	9225 (40.6)	6706 (40.7)	2519 (40.4)	
Pack-year of smoking, median (IQR)	39 (28-51)	38 (27-49)	43 (30-54)	<.001
Time since quitting, median (IQR), y^e^	6 (3-11)	7 (3-11)	6 (3-10)	.009
Attempt to quit smoking in the past year^f^				
No	3045 (20.3)	2231 (20.7)	814 (19.3)	.04
Yes	2176 (14.9)	1480 (14.0)	696 (17.5)	
Unknown	8267 (64.8)	6174 (65.4)	2093 (63.2)	
E-cigarette use				
Never	13 066 (55.4)	9457 (54.7)	3609 (57.2)	<.001
Current	1940 (9.3)	1523 (10.2)	417 (6.7)	
Former	7707 (35.4)	5611 (35.1)	2096 (36.1)	
Heavy alcohol use				
No	19 611 (89.4)	14 254 (89.0)	5357 (90.6)	.06
Yes	2463 (10.6)	1851 (11.0)	612 (9.4)	
BMI				
Normal or underweight (<25.0)	6909 (30.7)	4994 (30.2)	1915 (32.0)	.43
Overweight (25 to ≤30)	7835 (33.4)	5709 (33.5)	2126 (33.0)	
Obese (≥30)	7969 (35.9)	5888 (36.3)	2081 (35.0)	
No. of comorbidities^g^				
0	5472 (24.9)	4526 (28.5)	946 (14.9)	<.001
1	7035 (30.2)	5340 (31.4)	1695 (26.7)	
2	5140 (23.1)	3519 (21.6)	1621 (27.1)	
≥3	5066 (21.9)	3206 (18.5)	1860 (31.2)	
Routine check-up last year				
No	3937 (18.8)	3433 (22.2)	504 (9.6)	<.001
Yes	18 584 (81.2)	12 998 (77.8)	5586 (90.4)	
Self-reported general health				
Poor	7646 (34.6)	5302 (33.0)	2344 (39.1)	<.001
Good	15 006 (65.4)	11 249 (67.0)	3757 (60.9)	
Geographic region^h^				
West	4623 (16.0)	3561 (16.4)	1062 (14.9)	.008
Midwest	6981 (25.5)	5141 (25.4)	1840 (25.9)	
Northeast	3740 (16.4)	2549 (15.3)	1191 (19.5)	
South	7029 (41.1)	5112 (42.1)	1917 (38.6)	
Other	340 (0.9)	228 (0.9)	112 (1.0)	
Time since last LCS				
≤1 y	3472 (14.6)	0	3472 (54.7)	NA
>1 y	2513 (11.4)	0	2513 (42.9)	
Unknown	137 (0.6)	0	137 (2.4)	
No LCS	16 591 (73.3)	16 591 (100.0)	0	

Abbreviations: BMI, body mass index (calculated as weight in kilograms divided by height in meters squared); BRFSS, Behavioral Risk Factor Surveillance System; LC, lung cancer; LCS, lung cancer screening; NA, not applicable.

^a^ Individuals who ever had computed tomography scans of the chest area to check or screen for lung cancer.

^b^ Individuals aged 80 years or older were grouped as 1 category within the BRFSS dataset. Thus, we limited our data to individuals aged 50 to 79 years.

^c^ Other races included: American Indian or Alaska Native, Asian, Native Hawaiian or Other Pacific Islanders, and multiracial groups.

^d^ Any private included a plan purchased through an employer or union or private nongovernmental plan. For individuals younger than 65 years, public insurance included Medicare, Medigap, Medicaid, Children’s Health Insurance Program, military related health care, Indian Health Service, state sponsored health plan, or other government program. For individuals aged 65 years or older, other public insurance included all of the above except for Medicare.

^e^ Limited to individuals who previously smoked.

^f^ Limited to individuals who currently smoke.

^g^ Comorbidities included: coronary heart disease or myocardial infarction, stroke, asthma, chronic obstructive pulmonary disease, chronic kidney disease, diabetes, and arthritis.

^h^ A total of 50 States and the District of Columbia, plus 3 US territories were included (eMethods in Supplement 1).

**Table 2.** E-Cigarette Use and LCS Uptake Among Eligible Individuals According to the USPSTF 2021 LCS Criteria^a^

Variable	Unweighted No.	Weighted % of uptake (95% CI)	*P* value	OR (95% CI)
**Had LCS in the past**				
Overall				
Never used e-cigarette	3609	27.5 (26.0-29.1)	<.001	1 [Reference]
Currently uses e-cigarette	417	19.4 (15.8-23.1)		0.79 (0.62-1.00)^b^
Previously used e-cigarettes	2096	27.2 (25.3-29.1)		1.03 (0.90-1.17)^b^
Currently smokes cigarettes	3603	26.8 (25.3-28.3)	NA	NA
Never used e-cigarettes	2060	27.6 (25.5-29.7)	.10	1 [Reference]
Currently uses e-cigarettes	207	20.8 (15.1-26.6)		0.80 (0.58-1.10)^c^
Previously used e-cigarettes	1336	26.8 (24.5-29.1)		1.02 (0.86-1.21)^c^
Previously smoked cigarettes	2519	26.5 (24.8-28.3)	NA	NA
Never used e-cigarettes	1549	27.5 (25.1-29.8)	.001	1 [Reference]
Currently uses e-cigarettes	210	18.2 (13.7-22.7)		0.73 (0.52-1.04)^d^
Previously usesd e-cigarettes	760	28.0 (24.8-31.3)		0.98 (0.80-1.20)^d^
**Up-to-date with LCS (within the past 1 y)**				
Overall				
Never used e-cigarettes	2072	15.4 (14.1-16.7)	<.001	1 [Reference]
Currently uses e-cigarettes	235	9.5 (7.5-11.4)		0.67 (0.51-0.88)^b^
Previously used e-cigarette	1165	14.7 (13.1-16.2)		0.96 (0.82-1.13)^b^
Currently smokes cigarettes	2048	14.3 (13.1-15.4)	NA	NA
Never used e-cigarettes	1196	15.1 (13.5-16.8)	.05	1 [Reference]
Currently uses e-cigarette	121	10.7 (7.6-13.8)		0.76 (0.52-1.12)^c^
Previously used e-cigarettes	731	13.8 (12.0-15.5)		0.91 (0.75-1.10)^c^
Previously smoked cigarettes	1424	15.1 (13.6-16.5)	NA	NA
Never used e-cigarettes	876	15.8 (13.8-17.8)	<.001	1 [Reference]
Currently uses e-cigarettes	114	8.3 (6.0-10.7)		0.54 (0.37-0.80)^d^
Previously used e-cigarettes	434	16.3 (13.5-19.1)		0.97 (0.75-1.25)^d^

Abbreviations: LCS, lung cancers screening; NA, not applicable; OR, odds ratio; USPSTF, US Preventive Services Task Force.

^a^ Stratified analysis to examine the impact of e-cigarette use on LCS behavior among all eligible individuals, as well as by their combustible smoking status.

^b^ Model adjusted for age, sex, race and ethnicity, education, income, pack-year of smoking, heavy drinking, body mass index (calculated as weight in kilograms divided by height in meters squared), number of comorbidities, insurance type, routine check-up within the past year, general heath, and region.

^c^ Model c = Model b + attempt to quit smoking in the past year.

^d^ Model d = Model b + year since quitting.

## Discussion

In this cross-sectional study, e-cigarette use was independently associated with lower use of LCS, particularly among individuals who had quit smoking combustible cigarettes. Emerging research suggests that e-cigarettes contain definite and probable carcinogens and cause similar cancer-associated gene deregulations as combustible tobacco.^4^ However, it has been shown that two-thirds of individuals currently using e-cigarettes consider e-cigarettes to be less harmful than combustible cigarettes.^3^ Thus, individuals who use e-cigarettes may have lower awareness of lung cancer risks. Limitations of this study include cross-sectional study design, self-reported smoking and LCS information, and inability to assess the impact of switching between cigarettes and e-cigarettes on LCS uptake. Our results highlighted the importance of raising awareness and rectifying misconceptions of e-cigarette use. Former smokers who use e-cigarettes remain at increased risk of lung cancer and should be targeted by interventions to improve adherence to LCS.

## Notes

Supplement 1.
eMethods.
**eFigure.** Participants Selection Flow Chart **eTable.** Calculation of Smoking History Variables
eReferences.

Supplement 2.
Data Sharing Statement
