# Supplementary material for: E-Cigarette Use and Lung Cancer Screening Uptake
Source: JAMA Netw Open. 2024 Jul 2;7(7):e2419648. doi: 10.1001/jamanetworkopen.2024.19648 (PMC11220562; doi:10.1001/jamanetworkopen.2024.19648)
Supplement: Supplement 1. — eMethods. eFigure. Participants Selection Flow Chart eTable. Calculation of Smoking History Variables eReferences. [file jamanetwopen-e2419648-s001.pdf]

Supplemental Online Content

Wang Q, Jiang C, Hsu ML, et al. E-cigarette use and lung cancer screening uptake. *JAMA Netw Open*. 2024;7(7):e2419648. doi:10.1001/jamanetworkopen.2024.19648

eMethods

eFigure. Participants Selection Flow Chart

eTable. Calculation of Smoking History Variables

eReferences.

This supplemental material has been provided by the authors to give readers additional information about their work.

© 2024 Wang Q et al. *JAMA Network Open.*

eMethods

Study sample

In a cross-sectional design, adults aged between 50 to 79 years old with available information regarding lung cancer screening (LCS) were identified from the Centers for Disease Control and Prevention's 2022 Behavioral Risk Factor Surveillance System (BRFSS) survey. The BRFSS survey is a health-related telephone survey that collects state data about US residents regarding health-related risk behaviors, chronic health conditions, and use of preventive services (eFigure 1).(1) A total of 50 States and the District of Columbia, plus 3 US territories were included in the analysis: West (Alaska, Arizona, California, Colorado, Hawaii, Idaho, Montana, Nevada, New Mexico, Oregon, Utah, Washington, and Wyoming), Midwest (Illinois, Indiana, Iowa, Kansas, Michigan, Minnesota, Missouri, Nebraska, North Dakota, Ohio, South Dakota, and Wisconsin), Northeast (Connecticut, Maine, Massachusetts, New Hampshire, New Jersey, New York, Pennsylvania, Rhode Island, and Vermont), South (Alabama, Arkansas, Delaware, District of Columbia, Florida, Georgia, Kentucky, Louisiana, Maryland, Mississippi, North Carolina, Oklahoma, South Carolina, Tennessee, Texas, Virginia, West Virginia), and Other US territories (Guam, Puerto Rico, Virgin Islands). The study was exempt for human participants review from the institutional review board at University Hospitals.

Measures

Individuals aged 80 years were not selected because in the BRFSS database, the variable _AGE80=80 was coded to represent individuals who were aged 80 and above. The question "(Ever told) (you had) melanoma or any other types of cancer?" was used to exclude individuals who had a prior history of cancer.

The question "Would you say you have never used e-cigarettes or other electronic vaping products in your entire life or now use them every day, use them some days, or used

© 2024 Wang Q et al. *JAMA Network Open.*

them in the past but do not currently use them at all?" was used to define e-cigarette use. For our analysis, we coded the e-cigarette use variable to never e-cigarette user = "Never used e-cigarettes in your entire life" response; current e-cigarette user = "Use them every day" or "Use them some days" response; and former e-cigarette user ="Not at all (right now)" response. Smoking (i.e., combustible cigarette) history variables were listed in eTable1. The United States Preventive Services Taskforce (USPSTF) 2021 eligibility was defined as individuals who ever smoked and aged between 50 to 80 years, and smoked at least 20 pack-years, currently smokes, or quit within the past 15 years. Those who met the USPSTF 2021 criteria were included. (2)

We used the questions "Have you ever had a CT or CAT scan of your chest area?" and "Were any of the CT or CAT scans of your chest area done mainly to check or screen for lung cancer?" to define LCS uptake. If an individual responded "Yes" to both questions, LCS uptake was defined as yes. In addition, if the response was "Within the past year (anytime less than 12 months ago)" to the question "When did you have your most recent CT or CAT scan of your chest area mainly to check or screen for lung cancer?", it was defined as having an up-to-date LCS.

Statistical Analysis

χ^2 tests (for categorical variables) and Kruskal-Wallis (for continuous variables) tests were used to compare the baseline demographic features and smoking behaviors between eligible individuals who had LCS vs did not have LCS (ever). Logistic regression model was used to calculate the association between LCS uptake (ever and up-to-date) and e-cigarettes use, overall and by combustible smoking status, after adjusting for potential confounders including age, sex, race/ethnicity (Hispanic, non-Hispanic Black, non-Hispanic White, and other [American Indian/Alaskan Native, Asian, Native Hawaiian/Other Pacific Islanders, Multiracial groups]), educational achievement, income (<50,000 USD, ≥ 50,000 USD vs unknown), pack-

© 2024 Wang Q et al. *JAMA Network Open.*

year of smoking (20-29, 30-39, vs 40+ pack-years), heavy drinking (yes vs no), body mass index (BMI) (normal/underweight, overweight, vs obese), number of comorbidities (0, 1 , 2 vs ≥ 3), insurance type (any private [<65 y], Public [<65 y], Medicare [≥ 65 y], Any private [≥ 65 y], Other public [≥ 65 y] and Uninsured), routine check-up within the past year (yes vs no), self-reported general health (good [excellent, very good or good] vs poor [fair or poor]), and region where the participants were located (West, Midwest, Northeast, South vs other). We additionally adjusted for attempt to quit smoking (yes vs no) when the analysis was limited to current smokers, and year-since-quitting (≤ 5, 6-10 vs 11-15 years) when limited to former smokers, respectively.

All calculations were weighted according to the BRFSS guidelines.(3) Listwise deletions were used to treat missing values across these variables.(4) The analysis was conducted using SAS 9.4 (SAS Institute Inc) and R software version 2024.04.1. The significance level was set at a 2-sided p<0.05. We followed the Strengthening the Reporting of Observational Studies in Epidemiology (STROBE) reporting guideline to ensure comprehensive and transparent reporting of our study.

© 2024 Wang Q et al. *JAMA Network Open.*

eFigure. Participants Selection Flow Chart

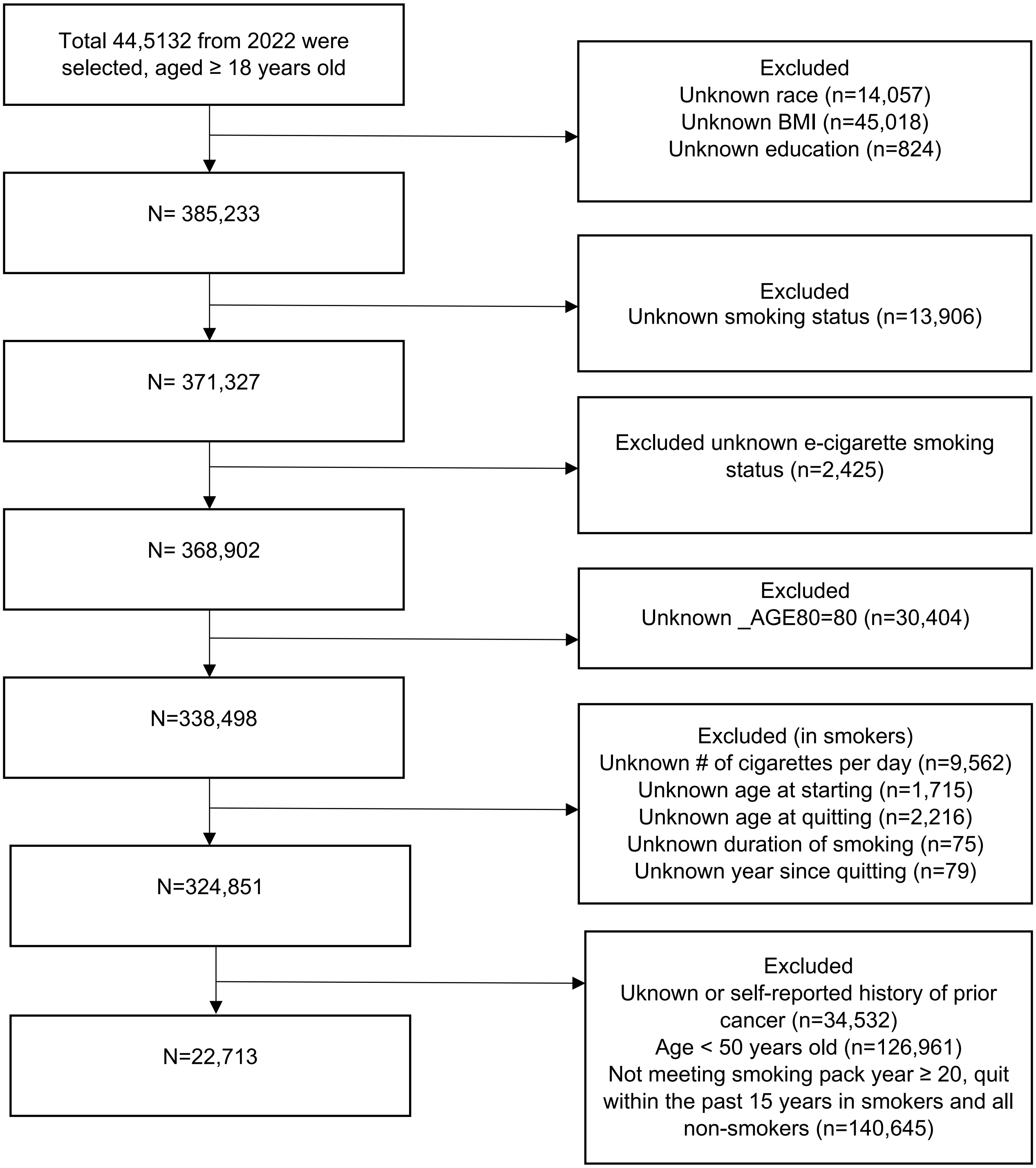

© 2024 Wang Q et al. *JAMA Network Open.*

eTable. Calculation of Smoking History Variables

Final Variable	Questions in the survey	Calculation	Description
LCSFIRST	How old were you when you first started to smoke cigarettes regularly?	NA	Age at starting
LCSLAST	How old were you when you last smoked cigarettes regularly?	NA	Age at quitting
PACK	On average, when you (smoke/smoked) regularly, about how many cigarettes (do/did) you usually smoke each day? (LCSNUMCG)	PACK=LCSNUMCG/20	# of Pack smokes/smoked per day
YEARQUIT	Imputed Age value collapsed above 80 (_AGE80)	Former smokers: YEARQUIT=LCSLAST - _AGE80	Year since quitting
YR	Imputed Age value collapsed above 80 (_AGE80)	Former smokers: YR=LCSLAST - LCSFIRST;<br><br>Current smokers: YR=_AGE80 - LCSFIRST	Duration of smoking
PY	On average, when you {smoke/smoked} regularly, about how many cigarettes {do/did} you usually smoke each day?<br><br>+ Duration of Smoking	PY=YR * PACK	Pack year of smoking
STOPSMK2	During the past 12 months, have you stopped smoking for one day or longer because you were trying to quit smoking? (current smokers)	Yes/No	Attempt to quit smoking in current smokers

© 2024 Wang Q et al. *JAMA Network Open.*

eReferences

1. Centers for Disease Control and Prevention (CDC). Behavioral Risk Factor Surveillance System Survey Data. Atlanta, Georgia: U.S. Department of Health and Human Services, Centers for Disease Control and Prevention. Available at: https://www.cdc.gov/brfss/index.html. Accessed May 1, 2023. .
2. U.S. Preventive Services Task Force. Lung Cancer Screening. Available at https://www.uspreventiveservicestaskforce.org/uspstf/recommendation/lung-cancer-screening. Accessed in Apirl 10, 2023.
3. BRFSS Weighting the Data 2022. Available at https://www.cdc.gov/brfss/annual_data/2022/pdf/2022-Weighting-Description-508.pdf. Accessed Janurary 20, 2024.
4. Maki KG, Tan NQP, Toumazis I, Volk RJ**.** Prevalence of Lung Cancer Screening Among Eligible Adults in 4 US States in 2021. JAMA Netw Open. 2023;6(6):e2319172.

© 2024 Wang Q et al. *JAMA Network Open.*
