# Supplementary material for: E-Cigarette Use and Lung Cancer Screening Uptake
Source: JAMA Netw Open. 2024 Jul 2;7(7):e2419648. doi: 10.1001/jamanetworkopen.2024.19648 (PMC11220562; doi:10.1001/jamanetworkopen.2024.19648)
Supplement: Supplement 2. — Data Sharing Statement [file jamanetwopen-e2419648-s002.pdf]

Data Sharing Statement

Wang. E-Cigarette Use and Lung Cancer Screening Uptake. *JAMA Netw Open*. Published July 02, 2024. doi:10.1001/jamanetworkopen.2024.19648

Data
Data available: Yes
Data types: Deidentified participant data
How to access data: The data and data dictionary are publicly available at CDC BRFSS data https://www.cdc.gov/brfss/index.html. The analytic code is available upon request to the corresponding author Dr. Qian Wang @qian.wang@uhhospitals.org
When available: With publication

Supporting Documents
Document types: Statistical/analytic code
How to access documents: The data and data dictionary are publicly available at CDC BRFSS data https://www.cdc.gov/brfss/index.html. The analytic code is available upon request to the corresponding author Dr. Qian Wang @qian.wang@uhhospitals.org
When available: With publication

Additional Information
Who can access the data: The data and data dictionary are publicly available at CDC BRFSS data https://www.cdc.gov/brfss/index.html. The analytic code is available upon request to the corresponding author Dr. Qian Wang @qian.wang@uhhospitals.org
Types of analyses: The data and data dictionary are publicly available at CDC BRFSS data https://www.cdc.gov/brfss/index.html. The analytic code is available upon request to the corresponding author Dr. Qian Wang @qian.wang@uhhospitals.org
Mechanisms of data availability: The data and data dictionary are publicly available at CDC BRFSS data https://www.cdc.gov/brfss/index.html. The analytic code is available upon request to the corresponding author Dr. Qian Wang @qian.wang@uhhospitals.org
